# Draft Genome Sequence of *Rheinheimera* sp. F8, a Biofilm-Forming Strain Which Produces Large Amounts of Extracellular DNA

**DOI:** 10.1128/genomeA.00082-16

**Published:** 2016-03-10

**Authors:** Anna-Kathrin Schuster, Ulrich Szewzyk

**Affiliations:** Technische Universität Berlin, Berlin, Germany

## Abstract

*Rheinheimera* sp. strain F8 is a biofilm-forming gammaproteobacterium that has been found to produce large amounts of filamentous extracellular DNA. Here, we announce the *de novo* assembly of its genome. It is estimated to be 4,464,511 bp in length, with 3,970 protein-coding sequences and 92 RNA-coding sequences.

## GENOME ANNOUNCEMENT

*Rheinheimera* sp. strain F8 is an aquatic biofilm-forming bacterium, which was isolated from “river snow” of the South Saskatchewan River, Saskatoon, Saskatchewan, Canada (1). F8 is a typical rod-shaped Gram-negative gammaproteobacterium about 2 µm in length and 1 µm in width, with nonpigmented to yellowish colonies. It was shown that F8 forms stable filaments of extracellular DNA, which have a width of 1.8 to 2.0 nm (2) and can be stained by DNA-specific fluorescent dyes, like Syto9 or propidium iodide (Molecular Probes, USA) (1).

An assessment of the metabolic profile of F8 using the commercial multiwell system Biolog (Biolog, Hayward, CA) showed that the strain is not fastidious. It was able to use the majority of carbohydrates and carboxylic acids offered, as well as a wide range of other substrates.

For the extraction of genomic DNA, F8 was grown on FBM agar plates at room temperature and harvested after 7 days. FBM medium contains 3.0 g of Na_2_SO_4_, 0.4 g of MgCl_2_·6H_2_O, 1.2 g of NaCl, 0.3 g of NH_4_Cl, 0.3 g of KCl, and 0.15 g of CaCl_2_·2H_2_O (pH 6,8) and was supplemented with yeast extract (75 mg/liter), glucose (10 µM), and a trace element (SL8) and vitamin solution.

Here, we report the *de novo* genome assembly of F8. It is the latest of eight fully sequenced strains in the genus *Rheinheimera*. The extraction and purification of genomic DNA were done with the bacterial and yeast genomic DNA kit (Roboklon, Germany). For library preparation, the TruSeq DNA LT library and Nextera mate-pair library version 2 prep kits (Illumina, United States) were used. Paired-end sequencing (2 × 300 bp) was performed using the Illumina MiSeq sequencer with the MiSeq reagent kit version 3. The resulting 7,192,490 reads were assembled with the CLC Workbench 8 and SPAdes 3.5.0. In the final assembly of the draft genome of F8, a 4,464,511-bp sequence with 3,970 protein-coding sequences, 9 genes for rRNAs (including 3 versions for 16S rRNA), and 83 genes for tRNAs was generated. The G+C content is 51.8%.

The 16S rRNA genes of 16 *Rheinheimera* strains were aligned with MEGA6, based on Clustal W, and phylogenetic analysis was made using the maximum-likelihood neighbor-joining algorithms. Those analyses suggest that *Rheinheimera tilapiae* (accession no. HQ111524 [3]) is the next relative of F8. Alignment with the NCBI Nucleotide Basic Local Alignment Tool (BLAST) shows an identity of 99% between the 16S rRNA genes of the two species.

### Nucleotide sequence accession number.

The draft genome sequence of *Rheinheimera* sp. F8 has been deposited at DDBJ/ENA/GenBank under the accession no. CP013656. The version described in this paper is the first version.
